# An Emerging Role for isomiRs and the microRNA Epitranscriptome in Neovascularization

**DOI:** 10.3390/cells9010061

**Published:** 2019-12-25

**Authors:** Reginald V.C.T. van der Kwast, Paul H.A. Quax, A. Yaël Nossent

**Affiliations:** 1 Department of Surgery and Einthoven Laboratory for Experimental Vascular Medicine, Leiden University Medical Center, 2333 ZA Leiden, The Netherlands; 2 Department of Laboratory Medicine and Department of Internal Medicine II, Medical University of Vienna, 1090 Vienna, Austria

**Keywords:** microRNA, isomiRs, epitranscriptome, neovascularization, angiogenesis, arteriogenesis, A-to-I editing, m6A, RNA modifications, RNA methylation

## Abstract

Therapeutic neovascularization can facilitate blood flow recovery in patients with ischemic cardiovascular disease, the leading cause of death worldwide. Neovascularization encompasses both angiogenesis, the sprouting of new capillaries from existing vessels, and arteriogenesis, the maturation of preexisting collateral arterioles into fully functional arteries. Both angiogenesis and arteriogenesis are highly multifactorial processes that require a multifactorial regulator to be stimulated simultaneously. MicroRNAs can regulate both angiogenesis and arteriogenesis due to their ability to modulate expression of many genes simultaneously. Recent studies have revealed that many microRNAs have variants with altered terminal sequences, known as isomiRs. Additionally, endogenous microRNAs have been identified that carry biochemically modified nucleotides, revealing a dynamic microRNA epitranscriptome. Both types of microRNA alterations were shown to be dynamically regulated in response to ischemia and are able to influence neovascularization by affecting the microRNA’s biogenesis, or even its silencing activity. Therefore, these novel regulatory layers influence microRNA functioning and could provide new opportunities to stimulate neovascularization. In this review we will highlight the formation and function of isomiRs and various forms of microRNA modifications, and discuss recent findings that demonstrate that both isomiRs and microRNA modifications directly affect neovascularization and vascular remodeling.

## 1. Introduction

Ischemic cardiovascular disease (CVD) is the leading cause of death in worldwide and was responsible for approximately 17.8 million deaths in 2017 [1,2]. Additionally, it is estimated that current standard therapies are unsuitable or insufficient for 30% of patients [3,4]. Therefore, there is a critical need for new therapeutic treatments for ischemic CVD. 

A potential strategy to treat patients with ischemia is to stimulate neovascularization, which is the body’s natural repair mechanism to restore blood flow to ischemic tissues. Postnatal neovascularization is comprised of angiogenesis, the sprouting of new capillaries from existing vessels, and arteriogenesis, the maturation of preexisting collateral arterioles into fully functional arteries. Both angiogenesis and arteriogenesis are highly multifactorial processes that involve multiple types of vascular and immune cells. In order to improve neovascularization as a whole, therapeutic strategies which simultaneously target both angiogenesis and arteriogenesis are needed [5,6]. 

During the last decade, microRNAs have emerged as multifactorial regulators of neovascularization [7,8,9]. MicroRNAs are short non-coding RNAs of approximately 22 nucleotides that inhibit translation of messenger RNAs (mRNAs). A single microRNA can have hundreds of mRNAs in its ‘targetome’, often regulating an entire network or pathway simultaneously [10]. MicroRNAs are typically defined as one specific sequence of RNA nucleotides, however, recent studies have shown that this ‘canonical’ microRNA sequence is often altered. These microRNA alterations can be grouped into two types: (i) isomiRs, which are microRNAs with altered terminal sequences and (ii) biochemical modifications of specific nucleotides within microRNAs, which collectively are referred to as the microRNA epitranscriptome. Both types of microRNA variations appear actively regulated in response to ischemia and can directly influence neovascularization associated processes, as we will discuss below. The microRNA epitranscriptome unveils a whole new regulatory layer that could provide novel therapeutic options for ischemic CVD. In this review we will first briefly introduce the processes involved in angiogenesis and arteriogenesis, after which we will highlight various ways in which a microRNA can be altered, and discuss recent findings which demonstrate that these microRNA alterations can affect neovascularization associated processes.

## 2. Neovascularization—Angiogenesis and Arteriogenesis

After the occlusion of a large artery, blood flow to the downstream tissues is hampered, causing ischemia. Blood flow towards the ischemic tissue can be restored by a process called arteriogenesis. Arteriogenesis is the growth and maturation of collateral arteries from a pre-existing arteriole network, which connects all major arteries in the body [5]. Arteriogenesis is triggered by an increase in shear stress in the arterioles, which occurs after an arterial occlusion causes redirection of blood flow through the arterioles. The increased shear stress causes endothelial cells (ECs) in the arteriole wall to express adhesion molecules and secrete cytokines, leading to the attraction of circulating monocytes and other immune cells [11,12,13,14,15]. These inflammatory cells produce and secrete proteases, growth factors, and cytokines which enable remodeling of the vessel wall and stimulate migration and proliferation of vascular ECs and smooth muscle cells (SMCs) [16,17,18]. This results in an increase in vessel diameter, until fluid sheer stress decreases which halts the arteriogenic process. Finally, the vascular SMCs and fibroblasts secrete matrix components to reconstitute the vessel wall [19,20].

The process of angiogenesis, on the other hand, is the sprouting of a new capillary from the existing vasculature in order to redistribute local blood flow towards ischemic areas. Unlike arteriogenesis, angiogenesis is driven by the hypoxia caused by ischemia, and revolves around resolving local ischemia rather than restoring arterial blood flow after the occlusion of a vessel. Angiogenesis is initiated when an angiogenic stimulus, produced by hypoxic cells, activates the vascular endothelial layer. Activated ECs will start to proliferate and migrate towards the stimulus, such as vascular endothelial growth factor (VEGF), resulting in a new capillary [21]. Next to ECs, other cell types are important regulators of angiogenesis. Vascular SMCs, pericytes, fibroblasts and immune cells play key roles by supporting and modulating EC function and secreting the proangiogenic stimuli to start the process [22,23,24].

Since both angiogenesis and arteriogenesis are highly multifactorial processes, the simultaneous stimulation of both processes requires a multifactorial regulator, like microRNAs [7,8].

## 3. MicroRNAs

MicroRNAs are endogenous, small non-coding RNA molecules that inhibit translation of mRNAs. The microRNA’s target selection is predominantly determined by the microRNA’s ‘seed sequence’, nucleotides 2–8 at the 5′-end of a microRNA [25,26], which bind their target mRNAs via Watson–Crick base-pairing. Due to this relatively small targeting sequence, a single microRNA’s ‘targetome’ can consist of hundreds of mRNAs, enabling microRNAs to regulate multifactorial processes [10].

**Figure 1:**
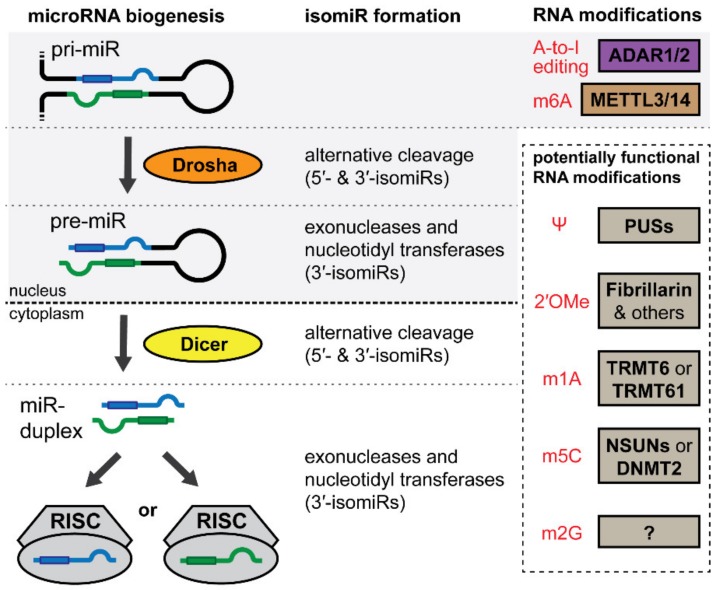
MicroRNA biogenesis and alterations that induce isomiR formation or microRNA nucleotide modifications. Transcription of the microRNA containing gene forms the primary microRNA (pri-miR). Drosha cleaves the pri-miR to generate the precursor microRNA (pre-miR). The pre-miR cleaved by Dicer in the cytoplasm yielding the microRNA duplex. Either side of the duplex can be incorporated into the RNA-induced silencing complex (RISC) to become a functional mature microRNA. IsomiRs can be formed during microRNA biogenesis when Drosha or Dicer cleave in alternative locations, or when exonucleases or nucleotidyl transferases remove or add nucleotides to the 3′-end of the pre-miR or the mature microRNA. RNA nucleotide modifications with known or potential functional implications on microRNA biogenesis or functioning are shown in red with their ‘writers’ next to them.

The biogenesis of microRNAs starts with the transcription of the microRNA containing gene, yielding a primary microRNA (pri-miR) which then undergoes several steps of maturation to form the mature and functional microRNA (Figure 1) [27]. First, the pri-miR is cleaved in the nucleus by Drosha to generate a hairpin-shaped precursor microRNA (pre-miR) [28]. The pre-miR is then translocated to the cytoplasm where a final cleavage is performed by Dicer, yielding a microRNA duplex [29]. Either side of the duplex can associate with Argonaute proteins and become a functional mature microRNA after incorporation into the RNA-induced silencing complex (RISC) [30]. Mature microRNAs are named after their side, 5′ or 3′, in the pri-miR hairpin (e.g., miR-#-5p or -3p).

MicroRNA biogenesis is strictly regulated, even at a microRNA-specific level, by numerous factors, including DNA methylation, activity modulation of key maturation proteins and many RNA-binding proteins [29,31,32,33]. As a result, microRNA expression is often highly tissue specific and is dynamically regulated during key physiological processes, including the response to ischemia [29,34].

In 2007, the importance of microRNAs in neovascularization was demonstrated for the first time when several studies showed that Dicer-dependent microRNAs were required for angiogenesis [35,36,37]. Since then, microRNAs have been shown to play a functional role in all processes involved in neovascularization, including production and secretion of angiogenic stimuli, as well as EC, SMC, fibroblast and immune cell proliferation, migration and activation, which have recently been reviewed in references [8,38,39,40]. Several of these vasoactive microRNAs have also been well described to play an important role in vascular remodeling during ischemic cardiovascular diseases [8,41].

For example, Bonauer et al. showed that miR-92a is highly expressed in human ECs and functions as negative regulator of angiogenesis [42]. Inhibition of miR-92a increased angiogenesis in vivo and improved blood flow recovery after hindlimb ischemia [42]. Furthermore, administration of miR-92a inhibitors in porcine models for myocardial infarction demonstrated that miR-92 inhibition prevents adverse infarct remodeling and ischemia/reperfusion injury [43,44]. Phase 1 trials aimed to improve wound healing with a future potential clinical application towards heart failure treatment have recently been completed for miR-92a inhibitor MRG 110 (NCT03603431) [45].

Both miR-126-3p and -5p are also highly expressed in ECs where they promote angiogenesis by stimulating EC proliferation and VEGF signaling and regulating leukocyte adhesion [46,47,48,49]. Inhibition of miR-126-3p was shown to decrease recovery after myocardial infarction and hindlimb ischemia in mice [47,50,51]. Furthermore, miR-126 levels are decreased in patients with ischemic coronary artery disease [52]. Similarly, miR-10a also stimulates angiogenesis by promoting VEGF signaling in ECs and regulating their inflammatory phenotype [53,54,55,56,57].

MiR-21-5p regulates proliferation and apoptosis of vascular wall smooth muscle cells [58,59] and promotes fibrosis by stimulating fibroblast survival and growth factor secretion [60]. Preclinical studies have shown that inhibition of miR-21-5p can prevent maladaptive vascular remodeling and heart failure [59,60]. These findings suggest that the miR-21-5p inhibitor RG-012, which is currently being tested in a phase 2 clinical trial to prevent kidney fibrosis in patients with Alport syndrome (NCT02855268), could potentially be used for the treatment of CVD.

Additionally, it is noteworthy that several groups of genomically clustered microRNAs have been identified that are able to broadly regulate neovascularization in response to ischemia: Knockout of the miR-17/92 gene cluster (located on chromosome 14 in mice and on human chromosome 13) increased both angiogenesis and arteriogenesis [61,62], while the inhibition of individual microRNAs from the 14q32 microRNA cluster (located on chromosome 12F1 in mice and on human chromosome 14) was shown to independently stimulate both angiogenesis and arteriogenesis [9]. 

## 4. IsomiRs and the microRNA Epitranscriptome

Typically, microRNAs have been defined as a single sequence of RNA nucleotides, and are listed as such in the principle public microRNA database, miRbase [63]. However, recent studies have shown that this ‘canonical’ microRNA sequence can be altered. These microRNA alterations can be separated into two types: isomiRs and RNA nucleotide modifications.

IsomiRs are microRNA sequence variants that have one or more nucleotides added or deleted at their 5′- and/or 3′-ends compared to the canonical microRNA sequence. 

RNA nucleotide modifications are biochemical modifications of the standard RNA nucleotides, which are performed by enzymes present in all living organisms. Recent studies have demonstrated that these RNA nucleotide modifications have a functional regulatory role and form what has been named the ‘epitranscriptome’ [64]. While many different RNA nucleotide modifications exist, only a few have been studied in the context of microRNAs: Adenosine-to-inosine editing (A-to-I editing) and N6-adenosine methylation (m6A) and 2′-*O*-methylation (2′OMe).

Below, we will discuss those studies that demonstrate that isomiRs and microRNA A-to-I editing and m6A can be actively regulated and play a directing role in neovascularization, as well as other modifications (including 2′OMe) that are likely to have a similar role.

## 5. IsomiRs

### 5.1. IsomiRs

IsomiRs were discovered when microRNA sequencing studies observed that many microRNAs had sequence variants with one or more nucleotides added or deleted from the 5′- and/or 3′-ends compared to the ‘canonical’ microRNA sequence [65,66]. While initially dismissed as errors or artifacts, isomiRs have since been shown to be functional microRNAs which actively associate with the RISC complex and inhibit mRNA translation of their targets [67,68,69,70]. Furthermore, sequencing studies have shown that isomiRs are widespread and represent approximately 50% of the microRNA transcripts present in cells and tissue [71,72].

IsomiRs are primarily generated by cleavage variations of either DROSHA or DICER during microRNA biogenesis (Figure 1) [68,73]. IsomiRs with altered 3′-end sequences, 3′-isomiRs, can also be created by exonucleases which remove 3′ nucleotides, or by nucleotidyl transferases, which catalyze the addition of 3′ nucleotides. The number and type of isomiRs that arise from a single locus varies per microRNA, but approximately 75% of microRNA loci give rise to at least one isomiR [74]. 

**Figure 2:**
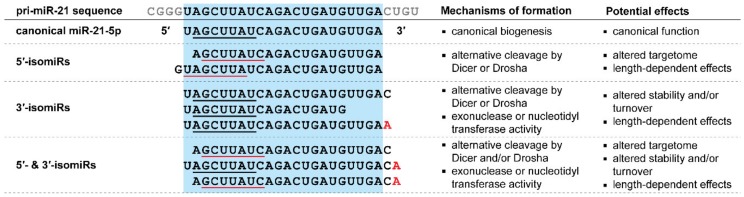
Different types of isomiRs, their mechanism of formation and their potential functional effects. The sequence of miR-21 and some of its isomiRs are shown to exemplify the different isomiR types. In each case, the seed sequence is underlined (red if altered) and red nucleotides are due to nucleotidyl transferase activity. Relative to the canonical microRNA, 5′-isomiRs generally have an altered targetome due to shift in seed sequence whereas 3′-isomiRs can affect the microRNAs stability or turnover. Both types of isomiRs affect the length of the microRNA and can thus incur length-dependent effects.

In general, 3′-isomiRs are more abundant than 5′-isomiRs, however, a number of microRNAs do have prevalent 5′-isomiRs [75,76,77,78]. Since a microRNA’s 5′-end determines its seed sequence, 5′-isomiRs have an altered targetome compared to the canonical microRNA sequence and are thus functionally different (Figure 2) [67,79,80,81]. While 3′-isomiRs do not have an altered seed sequence, their 3′-end variability has been associated with altered microRNA stability and turnover [82,83,84,85,86]. Furthermore, recent findings have shown that changes in microRNA length due to 3′-end variation can affect microRNA targeting strength and activity in specific cases [87,88]. Combined, these findings highlight the importance to take isomiRs into account during microRNA research.

### 5.2. IsomiRs in Neovascularization Associated Cells and Processes

Due to the prevalence of isomiRs, most microRNAs that are known regulators of neovascularization have isomiRs. In fact, the microRNA loci with the most known isomiRs are miR-21-5p (Figure 2) and miR-10a-5p, two microRNAs with well-established roles in vascular biology and neovascularization [53,54,55,56,57], which have at least 40 isomiRs each [74]. MiR-21-5p isomiRs were found to be highly expressed in endothelial cells, as well as, miR-126-5p and -3p and their isomiRs, which are also well-established vasoactive microRNAs [8,89,90,91]. Combined, the miR-21-5p and miR-126 transcripts accounted for almost 40% of the total endothelial microRNA transcripts detected, including at least two 5′-isomiRs with physiologically relevant abundance [89,91]. One of these studies reported that approximately 55% of the total microRNA transcripts detected in human umbilical vein endothelial cells (HUVECs) were in fact isomiRs originating from 230 distinct microRNA loci [89]. For 33 of these microRNA loci, the isomiR variant was the most abundant form, rather than the canonical sequence. Since isomiRs often have altered stability and turnover [83,84,85,86], these abundant isomiRs could help regulate vasoactive microRNA expression. Furthermore, abundant 5′-isomiRs are likely to be functionally important due to their altered seed sequence and thus targetome.

IsomiR expression profiles can vary based on cell type and in response to biological stimuli, including stimuli associated with neovascularization [75,76,78,92]. For example, Voellenke et al. examined isomiR expression of normoxic and hypoxic human umbilical vein endothelial cells (HUVECs) using deep sequencing [89]. While the study lacked the power to identify any statistically significant patterns, the authors did observe that hypoxic conditions altered isomiR expression. Furthermore, Nejad et al. demonstrated that treating fibroblasts with interferon-beta, a regulatory factor in both angiogenesis and arteriogenesis [93,94,95], specifically decreased expression of the longer 3′-isomiRs from 13 microRNA loci, while the shorter isomiRs were generally upregulated [96]. Among the regulated microRNAs was miR-222-3p, which has been shown to regulate angiogenesis and inflammation-mediated vascular remodeling [97,98]. Interestingly, the longer 3′-isomiRs of miR-222-3p (>22 nt) were previously found to increase apoptotic activity, whereas the shorter isomiRs did not, suggesting the altered 3′-isomiR profiles could also be functionally important [88]. However, the exact factors that mediate the isomiR-specific regulation remain to be uncovered. It is likely that, similar to canonical microRNA biogenesis, isomiR biogenesis is regulated by a multitude of factors, including factors which specifically regulate individual isomiRs [29,31,68].

We have recently performed a focused study on the 5′-isomiR of miR-411-5p from the vasoactive 14q32 microRNA cluster in order to collect direct evidence that isomiRs are actively regulated during ischemia-induced neovascularization [99]. We found that miR-411′s isomiR expression profile was tissue-specific and that canonical miR-411-5p was less abundant than its 5′-isomiR in human vascular ECs, fibroblasts and in whole human venous tissue. We discovered that the expression of the 5′-isomiR is decreased relative to canonical miR-411-5p expression in response to acute ischemia, both in cells and in a murine model for effective neovascularization after ischemia [99]. Strikingly, the relative 5′-isomiR expression was upregulated instead in ischemic veins from patients with critical limb ischemia due to peripheral artery disease (PAD). We demonstrated that the 5′-isomiR has a different targetome than the canonical miR-411-5p and inhibits translation of, among others, the pro-angiogenic Angiopoietin-1. Finally, we showed that the 5′-isomiR decreases vascular cell migration while the canonical miR-411-5p does not [99]. Combined these data show that isomiR formation is indeed a functional pathway, which is actively regulated during ischemia, with direct implications for neovascularization.

Table 1 presents a summary of the key studies that demonstrate the prevalence and importance of isomiRs.

## 6. Adenosine-to-Inosine Editing

### 6.1. Adenosine-to-Inosine Editing

A-to-I editing is the biochemical modification of adenosines into inosines by deamination. Unlike adenosine, inosine preferentially binds to cytidine and is therefore generally interpreted as guanosine by the cellular machinery [100]. This form of RNA editing can have a number of consequences on RNA functioning, ranging from destabilizing the RNA molecules’ secondary structure to altering a protein amino acid sequence due to editing of the mRNA’s coding sequence [101,102,103]. In mammals, A-to-I editing accounts for more than 90% of all RNA editing events and is catalyzed by either ADAR1 or ADAR2 (adenosine deaminase acting on RNA 1 or 2), which are expressed throughout the body [104,105,106]. The removal of the editing activity of either ADAR1 or ADAR2 in mice leads to premature lethality, demonstrating that A-to-I editing is of vital importance [107,108,109]. However, the precise regulatory mechanisms governing this critical cellular process have yet to be fully elucidated [110]. Changes to ADAR levels or its activity were shown to affect global editing, but these observations do not always correlate well with frequencies of individual editing events [111,112]. Therefore, it is evident that additional regulatory mechanisms exist that modulate A-to-I editing in a site-specific manner.

ADARs specifically target double stranded RNA structures, including those found in pri-miRs (Figure 1). The editing of a pri-miR can profoundly influence microRNA maturation, resulting in changes in mature microRNA expression [113,114,115]. However, when editing alters the microRNA’s seed sequence, this can completely change the mature microRNA’s target selection, resulting in the regulation of a different targetome [116].

### 6.2. MicroRNA A-to-I Editing in Neovascularization

MicroRNA editing is a widespread phenomenon which also affects many vasoactive microRNAs, as demonstrated recently in a study by Li et al. The authors mapped microRNA A-to-I editing at an unprecedented scale and found 2711 potential pri-miR editing sites within approximately 80% of all human pri-miRs [117]. MicroRNA editing profiles were also found to be tissue-specific, which is in agreement with previous findings [113,115,118]. Furthermore, 367 potential editing sites were found within human mature microRNAs, often located in the seed sequence [117]. 

In the field of cancer research, several microRNA editing events were shown to have a functional effect on cell migration and/or proliferation [119,120,121], which are also crucial processes in both angiogenesis and arteriogenesis [20,21]. For example, seed sequence editing of miR-455-5p was shown to alter its targetome, causing edited miR-455-5p to decrease tumor cell proliferation and migration, while the unedited version had the opposite effect [122]. Furthermore, editing of the seed sequence of miR-200b enhanced tumor cell proliferation and migration, in contrast to the unedited version [120]. Interestingly, higher miR-200b editing levels were associated with a poorer prognosis in cancer patients, highlighting the possibility that microRNA editing can be clinically relevant as a biomarker or therapeutic target. 

We have recently demonstrated that microRNA-editing can also directly regulate neovascularization. We showed that A-to-I-editing of miR-487b-3p, another microRNA from the vasoactive 14q32 microRNA cluster, is increased in ischemic muscle tissues undergoing neovascularization after induction of hindlimb ischemia [123]. MiR-487b-3p editing was also found in all human vascular ECs, SMCs, and fibroblasts. The edited mature miR-487b-3p has a unique targetome and promotes angiogenesis, in contrast to the canonical miR-487b-3p [123]. In a follow-up study, we demonstrate that vasoactive microRNA editing is a widespread phenomenon that enhances neovascularization in response to ischemia (manuscript submitted).

## 7. N6-Adenosine Methylation

### 7.1. N6-Adenosine Methylation

The modification of adenosine to N^6^-methyladenosine (m6A) is perhaps the most prevalent RNA nucleotide modification in eukaryotic cells and is present in more than 25% of human transcripts [124,125]. m6A is installed by the methyltransferase complex containing ‘writer’ METTL3 (Methyltransferase Like 3) and RNA-binding platform METTL14 [126], supported by cofactors WTAP (Wilms’ tumor 1-associating protein) and KIAA1429 [127,128]. Strikingly, m6A levels are dynamically regulated throughout all stages of life, with the help of m6A demethylases, or ‘erasers’, FTO (fat mass and obesity-associated protein), and ALKBH5 (alkB homolog 5) [129,130]. m6A methylation has been shown to affect almost every aspect of RNA metabolism, from expression and processing in the nucleus to translation and degradation in the cytoplasm [131,132,133]. The importance of its functions is illustrated by studies that demonstrate that individual knockout of either METTL3, METTL14 or WTAP causes prenatal lethality in mice [134,135,136]. While m6A can alter RNA folding and structure [137,138], most of m6A’s biological functions are mediated through a group of ‘reader’ proteins that specifically recognize the methylated adenosine on RNA, including the YTHD (YT521-B homology domain) and the IGF2BP (insulin-like growth factor-2 mRNA-binding protein) families [127,139,140,141].

While most m6A research has focused predominantly on mRNAs, several studies have demonstrated that m6A is important for microRNA biogenesis. Alarcon et al. demonstrated that pri-miRs are marked by the METTL3-dependent m6A (Figure 1). Pri-miR m6A marks are read by m6A-binding protein hnRNPA2B1 that, in turn, stimulates initiation of DICER-mediated processing through recruitment of DICER’s cofactor DGCR8 [142,143]. Intriguingly, a study by Berulava et al. demonstrated that well over 200 mature microRNAs contain m6A in a human embryonic kidney cell line (HEK293). While m6A does not affect canonical base pairing, several studies have suggested that it may block the noncanonical A:G base pairing, which could affect mRNA-microRNA interaction strength [138,144]. This is supported by a recent study that found that an m6A modified miR-200c-3p resulted in significantly less suppression of its target genes than unmethylated miR-200c-3p [145]. Furthermore, recent studies also suggest that m6A of mRNAs can influence their ‘targetability’ by microRNAs by promoting or preventing the binding of certain RNA-binding proteins that block microRNA-mediated transcript destabilization [141,146].

### 7.2. Importance of m6A in the Cardio-Vasculature and in Vasoactive MicroRNAs

Two recent studies have demonstrated the importance of m6A in cardiovascular homeostasis. Dorn et al. demonstrates that METTL3-dependant m6A helps modulate cardiac homeostasis and hypertrophic stress responses in mice [147]. The overexpression of METTL3 was shown to cause spontaneous hypertrophy, whereas METTL3 knockdown leads to maladaptive remodeling and signs of heart failure. Mathiyalagan et al. demonstrated that m6A is increased in failing mammalian hearts and in hypoxic cardiomyocytes [148]. Furthermore, increasing the expression of m6A eraser FTO in ischemic mouse hearts attenuates the ischemia-induced increase in m6A and decrease in cardiac contractile function. These findings highlight a key role for m6A in ischemic cardiovascular disease. 

Pri-miR m6A marks were shown to be required for the appropriate processing of most pri-miRs to mature miRNAs, including vasoactive microRNAs [142,143]. Furthermore, m6A of the above mentioned vasoactive miR-126 and miR-222 was shown to affect cell migration and/or proliferation in cancer cells. A study by Ma et al., demonstrated that the pri-miR of miR-126 undergoes METTL14-dependent m6A, which facilitates its processing to mature miR-126 [149]. Decreased METTL14-dependent m6A of pri-miR-126 led to the reduced expression of miR-126, which in turn increased cancer cell migration and invasion [149]. Han et al. showed that METTL3-dependant m6A of pri-miR-222 increases its maturation to mature miR-222, resulting in the reduction of PTEN, and ultimately leading to the proliferation of bladder cancer [150]. Furthermore, METTL3 was increased in bladder cancer and correlated with poor patient prognosis [150].

Combined, the abundance of m6A, its importance in microRNA biogenesis and functioning, and the dysregulation of m6A during ischemia and cardiovascular disease, suggest that m6A of microRNAs could play an important role in ischemic cardiovascular disease and neovascularization.

## 8. Other Modifications in the microRNA Epitranscriptome

As mentioned above, numerous other RNA nucleotide modifications exist, however, their presence and function in small RNAs (16–28 nucleotides long), which consist mostly of microRNAs [151], remains understudied [152]. An important reason for this is that conventional methods to detect RNA modifications are often unsuitable for small RNAs [152,153]. Recently, Lan et al. optimized a screening based on mass spectrometry which allowed them to provide the first overview of RNA nucleotide modifications in mammalian small RNAs using human HEK293T cells [154]. Besides inosine and m6A, 22 additional distinct nucleotide modifications were found, 13 of which consisted of different types or combinations of RNA methylations [154]. While little is known about the effect of these RNA nucleotide modifications on the functioning of small RNA, and thus microRNA, several have been studied in other RNA types. 

Below, we will report the key findings of these studied RNA modifications and highlight which properties could potentially affect microRNA function. Furthermore, the discussed nucleotide modifications and their potential effects on microRNAs are summarized in Table 2.

### 8.1. Pseudouridine (Ψ)

Pseudouridine (Ψ) is one of the most abundant RNA modifications [155,156]. Ψ is highly conserved and is generated from isomerization of uridine, catalyzed by pseudouridine synthases (PUSs) [155,156]. Recent advances in high-resolution detection methods have demonstrated that Ψ-nucleotides are found in many, if not all, species of RNA [156,157]. Pseudouridylation was shown to be important for ribosomal RNA biogenesis, pre-mRNA splicing, and translation fidelity [155,156]. Compared to a uracil, Ψ forms a stronger base pairing interaction with adenosine, which allows it to alter RNA secondary structures, suggesting that microRNA pseudouridylation could affect mRNA silencing [158,159]. Furthermore, transcriptome wide pseudouridylation was shown to increase under stress conditions, including serum deprivation, a key component of ischemia [160].

### 8.2. 2′-O-Methylnucleosides

It is known that 2′-*O*-methylation (2′OMe) can reside on all four ribonucleosides and is widely conserved [161,162]. Furthermore, 2′OMe is performed by methyltransferases like Fibrillarin and many, if not all, 2′OMe-events are directed by small nucleolar RNAs [64,163,164]. 2′OMe appears essential in processing ribosomal RNAs, small nuclear RNAs, and transfer RNAs, but it has also been found in mRNAs and even in microRNAs, by our group among others [123,161,165]. While the precise location and function of 2′OMe sites in many RNA types are currently unclear, 2′OMe in general has a stabilizing effect and can influence interactions with proteins or other RNAs [161,162]. 2′OMe may in fact protect adenosine residues from A-to-I editing [165,166,167]. Interestingly, we found that both 2′OMe and A-to-I editing of the same adenosine residue in pri-miR-487b are increased simultaneously under ischemia [123]. However, further studies are required to examine whether both RNA modifications can be found on a single copy of miR-487b-3p. Finally, 2′OMe also greatly enhances the stability of RNA-RNA duplexes, a quality that is often utilized to enhance the stability and specificity of synthetic antisense RNA-oligonucleotides, with similar implications for 2′OMe of microRNAs [168,169,170,171].

### 8.3. N1-Methyladenosine (m1A)

Recent methodological advances have demonstrated that m1A is a transcriptome-wide modification [172,173]. Several members of the TRMT family (tRNA methyltransferase family) have already been shown to be m1A writers and additional writers are thought to exist [172,173,174]. Similar to m6A, m1A is reversible and can be demethylated by erasers ALKBH1 and 3 (alkB homolog 1 and 3) [173,175]. Furthermore, m1A levels are dynamically regulated by various types of cellular stress and correlate with upregulation of translation in general [172,173]. This modification carries a positive charge and can therefore alter both protein–RNA interactions and RNA secondary structures dramatically [131], which can potentially lead to disruption of microRNA biogenesis [29]. Furthermore, m1A appears to disrupts RNA base-pairing and induces local RNA duplex melting, suggesting that m1A may also affect microRNA-target interactions [132,176].

### 8.4. N5-Methylcytosine (m5C)

While best known as a DNA modification in the epigenome, m5C can be installed on RNAs too by members from the NSUN family (nucleolar protein/sun RNA methyltransferase family) and by DNMT2 (DNA methyltransferase-2) and is therefore also part of the epitranscriptome [177,178,179,180,181]. m5C has been found in both noncoding and coding RNAs in mammals and a few studies have shown that m5C has functional implications [177,182,183]. For example, m5C of tRNAs was shown to protect tRNAs against stress-induced cleavage [180,184,185]. Furthermore, the depletion of m5C methyltransferase Nsun7 in mice resulted in a concomitant decrease of expression of specific non-coding RNAs, suggesting m5C marks can enhance RNA stability [186].

### 8.5. N2-Methylguanosine (m2G)

In tRNAs and rRNAs, m2G is a relatively common RNA modification, however, which m2G writers are responsible in humans remains unclear [187,188]. Interestingly, the study by Lan et al. demonstrated that m2G is also relatively common in small RNAs [154]. Our knowledge about this RNA modification is still very limited due to a lack of high-throughput detection methods [188,189]. However, studies have shown that m2G can form both canonical and non-canonical Watson–Crick base pairing interactions, allowing m2G to regulate the stability of tRNA tertiary structures and potentially influence microRNA silencing activity [188,190]. 

## 9. Dynamic Regulation of the Epitranscriptome

The epitranscriptome is dynamically regulated. This is abundantly clear for m6A modifications due to the discovery of both m6A writers and erasers [147,148]. Not all modifications may be reversible like m6A, but most, if not all, other modifications do appear to be regulated. Several studies have shown that RNA alterations are modulated under stress and pathological conditions [64,191,192]. For example, the deposition and distribution of m6A were increased in response to heat shock and DNA damage [193,194,195]. Total transcriptomic pseudourydilation increased in response to heat shock, nutrient deprivation, and serum deprivation [157,160]. Further, m1A levels in mammalian cells also increased in response to heat shock, but decreased after nutrient starvation [172]. Furthermore, cellular m5C levels are decreased in response external stress and cytotoxic stress which affects protein translation rates [196,197]. Additionally, the expression of methyltransferase Fibrillarin is increased in many cancers to facilitate additional 2′OMe of ribosomal RNAs [162,198,199], while mRNA A-to-I editing is induced by both hypoxia and inflammation [200]. Importantly, we have shown that both A-to-I editing and 2′OMe also increase in microRNAs during ischemia [123,201]. These findings suggest that the microRNA epitranscriptome is likely to also be dynamically regulated and functional in pathological conditions, and could provide novel targets for therapeutic intervention.

Several studies have also indicated that certain RNA nucleotide modifications regulate each other. As mentioned previously, 2′OMe was found to protect adenosine residues from A-to-I editing [165,166,167]. A different study demonstrated that replacing an adenosine which can be A-to-I edited by m6A also prevents editing almost completely in an in vitro assay [202]. Furthermore, it was recently demonstrated that transcript m6A levels are negatively correlated with the A-to-I editing levels of the transcript, even when they are not competing for the same nucleotide [203]. The depletion of m6A resulted in upregulated A-to-I editing on the m6A-depleted transcripts, confirming a transcriptome wide interplay between m6A and A-to-I editing [203]. These findings highlight the complexity of the epitranscriptome and the importance of studying multiple RNA modifications simultaneously in order to examine known interactions and to identify novel interactions.

## 10. Concluding Remarks

During the past decade, both isomiRs and the epitranscriptome have emerged as novel and dynamic layers of regulation of gene expression. Both types of microRNA alterations have been shown to modulate key physiological responses, including neovascularization by affecting the microRNA’s biogenesis, stability and function. MicroRNAs have already been established as multifactorial regulators of both angiogenesis and arteriogenesis [7,8,9], and therefore this additional regulatory layer may provide new options for therapeutic neovascularization. The therapeutic potential of both isomiRs and the microRNA epitranscriptome is highlighted by the findings that 5′-isomiR formation of miR-411-5p and A-to-I editing of miR-487b-3p are actively regulated in response to ischemia in vivo, resulting in novel microRNAs with anti- or pro-angiogenic properties, respectively [99,123]. Therefore, altered microRNAs could provide novel targets for therapeutic inhibition or overexpression to stimulate neovascularization after ischemic CVD.

The first therapeutic small RNA (Patisiran) was granted FDA approval in 2018 and the first phase 2 clinical trials with microRNA-oriented RNA therapeutics are currently ongoing, highlighting that microRNA therapeutics are on their way to clinical practice [45]. Over the last decade, important advances have been made in development of microRNA therapeutics, however, several key issues remain, which have been expertly reviewed in the studies by Lucas et al. and Rupaimoole et al. [41,204]. These issues include maximizing the effect of the therapeutics’ effect on the diseased tissue, while minimizing the off-target binding and toxicity. Now that the prevalence and functionality of microRNA alterations are becoming clear, further research is warranted to understand which altered microRNAs could pose off-target risks during design and development of microRNA-based therapeutics. However, uncovering the intricate mechanisms which govern regulation of microRNA alterations could also reveal novel therapeutic targets to modulate microRNA functioning.

Alternatively, tissue- and pathology-specific regulation of the microRNA alterations could potentially be used as a biomarker for cardiovascular disease, considering that isomiR expression profiles were found sufficient to distinguish between cancer subtypes [205].

Finally, while the abundance and function of many nucleotide modifications have not been studied in microRNAs yet, it is likely that most, if not all, will prove clinically relevant. The unique properties of certain nucleotide modifications, like for example m6A, could be exploited to enhance the specificity of microRNA-therapeutics when targeting microRNAs carrying such modifications. It is important to note that further advances in technology and methodology are required to expand our knowledge of the microRNA epitranscriptome [154,206]. However, given the surge of interest in this field, we expect many more clinically relevant microRNA alteration events to be discovered in the near future.

## Figures and Tables

**Table 1:** Key studies demonstrating the prevalence and importance of isomiRs.

Topic	Key Findings	References
**Prevalence of isomiRs**	Generally, isomiRs represent ~50% of microRNA transcripts in cells and tissues (~55% in HUVECs)	[71,72,89,91]
	~75% of microRNA loci can produce at least 1 isomiR	[74]
**Potential functional effect of isomiRs**	5′-isomiRs have altered targetome due to a shifted seed sequence compared to the cannonical microRNA	[67,79,80,81,99]
	3′-isomiRs can have altered microRNA stability and turnover	[82,83,84,85,86]
	isomiRs with different length can have altered targeting strength and activity	[87,88]
**Abundant vasoactive microRNAs with isomiRs**	miR-21-5p (at least 43 potential isomiRs, also in HUVECs)	[74,89,91]
	miR-10a-5p (at least 41 isomiRs)	[74]
	miR-126 (highly abundant in HUVECs together with miR-21-5p and its isomiRs)	[89,91]
	miR-222-3p (has functionally different 3′-isomiRs)	[88,96]
	miR-411-5p (5′-isomiR has altered functionality and anti-angiogenic properties)	[99]
**Regulation of isomiRs**	IsomiR expression profiles can vary based on cell type and in response to biological stimuli	[75,76,78,92]
	Hypoxic HUVECs display altered isomiR expression	[89]
	Independent regulation of miR-411-5p and its 5′-isomiR in response to ischemia	[99]

HUVECs: human umbilical vein endothelial cells.

**Table 2:** Known or postulated effects of nucleotide modification within microRNAs.

Nucleotide Modification	Abbreviation	Writers	Erasers	Potential Effects on microRNAs
Adenosine-to-inosine editing	A-to-I editing	ADAR1 or ADAR2	-	pri-miR editing can profoundly influence maturation seed sequence editing can alter targetome
N6-methyl-adenosine	m6A	METTL3/14	ALKBH5 FTO	regulates pri-miR processing hampered nonstandard A:G base pairing may affect silencing activity
Pseudouridine	Ψ	PUSs	-	stronger base pairing with adenosine might affect silencing activity *
2′-*O*-methyl-nucleosides	2′OMe	Methyl-transferases	-	may protect from A-to-I editing * may affect stability and turnover * enhanced RNA-RNA duplex stability might affect silencing activity *
N1-methyl-adenosine	m1A	TRMT6 & 61	ALKBH3	positive charge can dramatically alter interactions with proteins * disrupts RNA base pairing which can affect silencing activity *
N5-methyl-cytosine	m5C	NSUNs DNMT2	-	may enhance stability *
N2-methyl-guanosine	m2G	unclear	-	allows noncanonical base pairing which may affect silencing activity *

* effects are postulated effects based on observations in other RNA types.
